# Differences in the expression of caveolin-1 isoforms in cancer-associated and normal fibroblasts of patients with oral squamous cell carcinoma

**DOI:** 10.1007/s00784-021-03887-8

**Published:** 2021-03-27

**Authors:** S. Kaya, Nadine Wiesmann, J. Goldschmitt, M. Krüger, B. Al-Nawas, J. Heider

**Affiliations:** 1 Department of Oral and Maxillofacial Surgery Plastic Surgery, University Medical Center of the Johannes Gutenberg-University of Mainz, Augustusplatz 2, 55131 Mainz, Germany; 2 Molecular Tumor Biology, Department of Otorhinolaryngology, Head and Neck Surgery, University Medical Center of the Johannes Gutenberg-University, Langenbeckstraße 1, 55131 Mainz, Germany

**Keywords:** Oral squamous cell carcinoma, Fibroblasts, Cancer-associated fibroblasts, Caveolin-1α, Caveolin-1β

## Abstract

**Objectives:**

For many years, tumor development has been viewed as a cell-autonomous process; however, today we know that the tumor microenvironment (TME) and especially cancer-associated fibroblasts (CAFs) significantly contribute to tumor progression. Caveolin-1 (Cav-1) is a scaffolding protein which is involved in several cancer-associated processes as important component of the caveolae. Our goal was to shed light on the expression of the two different isoforms of Cav-1 in normal fibroblasts (NFs) and CAFs of patients with oral squamous cell carcinoma (OSCC).

**Materials and methods:**

Fibroblasts from normal mucosa and CAFs were isolated and propagated in vitro. Gene expression of the different Cav-1 isoforms was assessed via quantitative real-time PCR (qPCR) and supplemented by protein expression analysis.

**Results:**

We could show that the Cav-1β isoform is more highly expressed in NFs and CAFs compared to Cav-1α. Furthermore, the different Cav-1 isoforms tended to be differently expressed in different tumor stages. However, this trend could not be seen consistently, which is in line with the ambiguous role of Cav-1 in tumor progression described in literature. Western blotting furthermore revealed that NFs and CAFs might differ in the oligomerization profile of the Cav-1 protein.

**Conclusion:**

These differences in expression of Cav-1 between NFs and CAFs of patients with OSCC confirm that the protein might play a role in tumor progression and is of interest for further analyses.

**Clinical relevance:**

Our findings support a possible role of the two isoforms of Cav-1 in the malignant transformation of OSCC.

## Introduction

The oral squamous cell carcinoma (OSCC) is one of the six most common malignant tumors. With a worldwide cancer incidence of up to 355,000 new cases and a mortality of up to 177,000 deaths per year, the oral cavity is the most common location for squamous cell carcinoma of the head and neck region [1].

For years, tumor progression has been viewed as a cell-autonomous process and tumor research focused on the genetically mutated degeneration of tumor cells in the tumor process [2]. Today it is known that the interaction between tumor cells and cells of the tumor microenvironment (TME) is an important requirement for tumor progression. CAFs of the oral mucosa in OSCC are the most common cells in the TME [3]. It is believed that CAFs play an essential role in several processes of cancer biology including proliferation, invasion, angiogenesis, metastasis, and treatment resistance [4–6].

Caveolin-1 (Cav-1), a scaffold and structure protein, is expressed ubiquitously, with the highest Cav-1 levels being detected in endothelial cells, epithelial cells, adipocytes, fibroblasts, and CAFs [7]. However, the role of Cav-1 in tumor development varies in different tumor types [8] and is still subject of discussions. Although several studies have shown that Cav-1 can serve as a tumor oncogene, and induces tumor progression [9, 10] [11], other studies confirmed the role of Cav-1 as a tumor suppressor [12, 13]. Therefore, the role of Cav-1 in tumorigenesis remains unclear so far and Cav-1 appears to play a complex role in cancer development.

The protein Cav-1 is a member of the caveolin gene family [7] and is known as a structural component of caveolae, which are located on cellular membranes [14]. Caveolae and Cav-1 are involved in the vesicular transport via endocytosis, the extracellular matrix organization, the cholesterol distribution, and signal transduction [15, 16].

The Cav-1 protein is made up of 178 amino acids, structured into different domains. The C-terminal (amino acids 135-178) and the N-terminal (amino acids 1-101) end are facing towards the cytosol. Between the two terminal ends, the transmembrane region (amino acids 102-134) is located, which comes to lie within the plasma membrane. Other important domains are the oligomerization domain (amino acids 61-101) and the scaffolding domain (amino acids 82-101) [17].

**Fig. 1:**
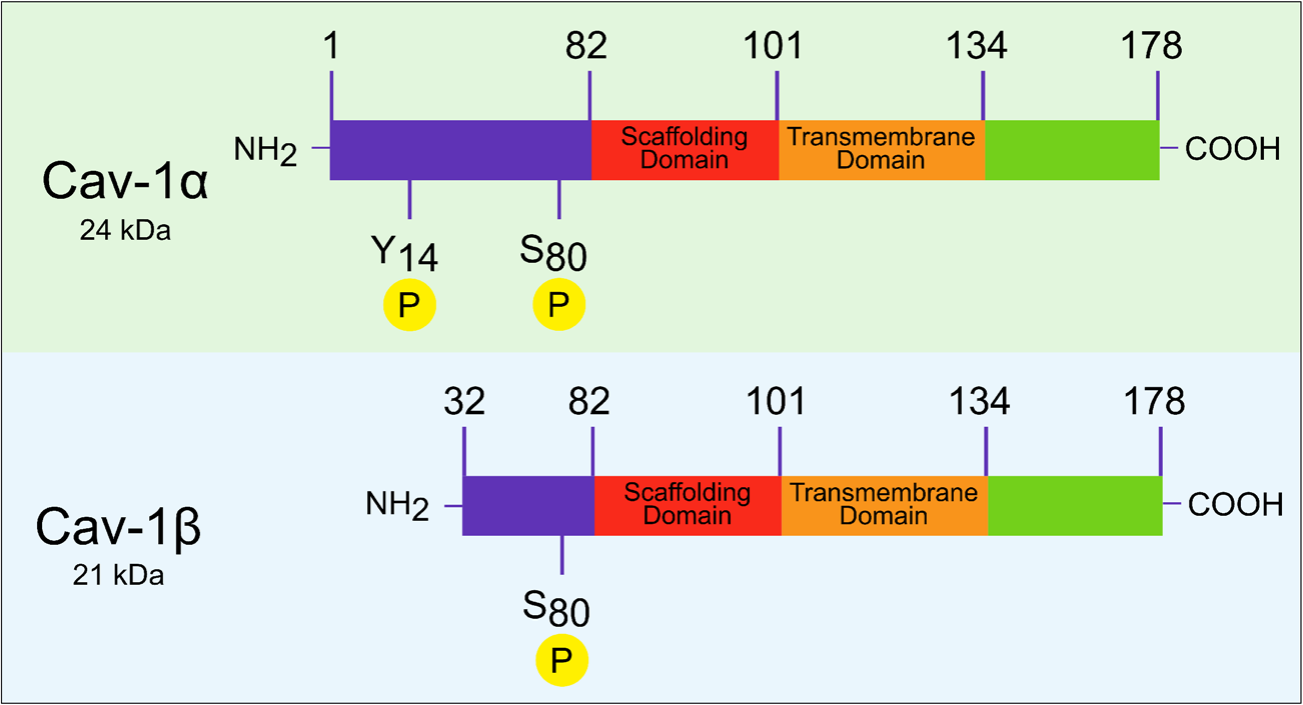
The molecular structure of the two isoforms of caveolin-1. NH_2_ = N-terminal end, COOH = C-terminal end, S = serine, Y = tyrosine, P = phosphorylation, the numbers indicate the position of the amino acid in the protein sequence

There are two isoforms of Cav-1, which differ in their molecular structure and are known as Cav-1α (24 kDa) and Cav-1β (21 kDa). Both isoforms share a complete C-terminal end, but they differ in their N-terminal end. In the protein sequence, only Cav-1α has a complete N-terminal end. Cav-1α is made up of 178 amino acids. In contrast, Cav-1β is 32 amino acids shorter at the N-terminal end (Fig. 1) [18]. Nevertheless, the two Cav-1 isoforms have a common hydrophobic stretch of amino acids, a framework domain, and an acetylated C-terminus [19]. So far, differences in the expression of these two isoforms in the fibroblasts of the oral mucosa have not been published.

Studies have shown that Cav-1α and Cav-1β might differ not only in their molecular structure but also in their function. Tyrosine phosphorylation only occurs at residue 14 (tyrosine 14) in the Cav-1α isoform [20, 21], which might result in different tasks of the isoforms within the cell [22]. Interestingly, Fujimoto et al. found that different types of caveolae formation exist. They showed the presence of shallow caveolae with different molecular compositions. Both isoforms of Cav-1 were present in the caveolae, but their ratio appeared to be crucial for the formation of the caveolae. Cav-1α was predominantly observed in deep caveolae, whereas Cav-1β was more prominent in shallow caveolae. The authors concluded that the ratio of Cav-1α to Cav-1β is higher in deep caveolae than in shallow ones [23].

The differences in the molecular structure of the two isoforms of Cav-1 could possibly explain the different roles that Cav-1 can play in tumor progression. The function of Cav-1 in tumor cells seems to be highly context-dependent exerting both tumor suppressive and oncogenic effects [8, 24]. Cav-1 was associated with tumor suppression, which is reflected in its ability to arrest the cell cycle [25, 26] and favor apoptosis [27]. On the other hand, Cav-1 was also shown to be involved in cancer cell migration and metastasis [28]. The individual functions of the two isoforms of Cav-1 are not fully elucidated in the context of tumor progression, yet. Thus, we wanted to shed light on their expression in CAFs.

We hypothesized that the ratio of the isoforms of Cav-1 to each other differs between normal fibroblasts and CAFs in patients with OSCC and that this shift in the expression of the isoforms in the tumor microenvironment may contribute to tumor development and progression. Therefore, the aim of the present study was to examine the expression of Cav-1α and Cav-1β in CAFs and normal fibroblasts (NFs) isolated from patients with OSCC and relate the expression profile of these two isoforms to the fibroblast type and the tumor staging. Current research shows clearly that more attention should be devoted to the tumor microenvironment of OSCC and that a more precise understanding of the tumor stroma can also benefit the treatment of tumor patients.

## Materials and methods

### Study design

In this prospective single-center pilot study, 20 patients with squamous cell carcinoma of the oral cavity (OSCC) who underwent tumor resection at the Department of Oral and Maxillofacial Surgery - Plastic Surgery at Johannes Gutenberg-University Mainz from 2015 to 2017 were included. Due to the pilot character of the study, there was no prior information upon which to base the sample size; thus, the achieved sample size is only based upon the number of patients that could be included in the study in the abovementioned period of time. Tumor and mucosal biopsies were taken from each patient. The biopsies from the mucosa were taken exclusively from the same patient from clinically healthy oral mucosa taken from the area furthest away from the respective resection margin. All patients signed a declaration of consent before participating in this study, and the ethics committee of the State Dental Association of Rhineland-Palatinate approved this study (ethics vote 837.387.11 (7929); year of ethics vote: 2011).

### Cell isolation and cell culture

CAFs from resected OSCC tissue and normal fibroblasts (NFs) from adjacent clinically healthy mucosa were isolated for cell culture. The tumor and mucosa biopsies were cut into small pieces of about 2 mm^3^ and sown in well plates with culture medium (Dulbecco’s modified Eagle medium with 10% fetal bovine serum (Gibco®, Life Technologies™, Paisley, UK), 1% penicillin-streptomycin-neomycin, 1% L-glutamine (Sigma-Aldrich Chemie GmbH, Steinheim, DE)) at 37°C and 5% CO_2_. The CAFs and NFs growing out of the tissue samples were further cultivated and used for the subsequent examination. Selection of fibroblasts from the samples was achieved by the chosen cell culture medium and rapid detachment of the cells with Accutase® Cell Detachment Solution during passaging. The growth of fibroblasts was determined phenotypically; we refrained from using any immunocytological markers, as CAFs in general [29] and CAFs of OSCC in particular [30] are highly heterogeneous populations and we did not want to miss any subpopulation.

### RNA extraction and quantitative real-time PCR

The isolation of the ribonucleic acid (RNA) from the CAFs and from the NFs was carried out with the RNeasy® Mini Kit (Qiagen GmbH, Hilden, Germany) according to the manufacturer’s protocol. The concentration and purity of the isolated RNA samples were determined with the NanoDrop® ND-1000 spectrophotometer (Peqlab Biotechnologie GmbH, Erlangen, Germany). The reverse transcription was performed using the peqstar thermal cycler (Peqlab Biotechnologie GmbH, Erlangen, Germany). The complementary deoxyribonucleic acid (cDNA) was synthesized from the messenger RNA (mRNA) using the enzyme reverse transcriptase. The qPCR for analysis of gene expression was performed with PCR Real-Time CFX Connect (Bio-Rad Laboratories GmbH, Hercules, CA, USA) and normalized to β-actin and glyceraldehyde 3-phosphate dehydrogenase (GAPDH) as reference genes. The primers for Cav-1α, Cav-1β, β-actin, and GAPDH were designed using the Primer-BLAST program and chosen as follows: Cav-1α (NM_001753.4) forward, 5′-CAGAACAAACCTTTGGCGGG-3′ and reverse, 5′-CCTTCCTGGGCATGGAGTCCT-3′; Cav-1β (NM_001172896.1) forward, 5′-TCGGAGCGGTTAGTTCGATT-3′ and reverse, 5′-GGTTGACCAGGTCGATCTCC-3′; β-actin forward, 5′-GGAGCAATGATCTTGATCTT-3′ and reverse, 5′-CCTTCCTGGGCATGGAGTCCT-3′; GAPDH forward, 5′-AAAAACCTGCCAAATATGAT-3′ and reverse, 5′-CAGTGAGGGTCTCTCTCTTC-3′.

### Protein extraction and immunoblotting

Cells were lysed on ice in RIPA buffer supplemented with protease inhibitor cocktail and phosphatase inhibitor cocktail 2 and 3 according to manufacturer’s recommendations (Sigma-Aldrich St. Louis, MO, USA). Samples were immediately quick frozen in liquid nitrogen and stored at −80°C. Prior to determination of protein concentrations (Pierce BCA Protein Assay Kit, Thermo Scientific, Waltham, MA, USA), lysates were clarified by centrifugation at 8000×*g* for 10 min at 4°C to pellet the debris.

Fifteen micrograms of total cellular protein of each sample was separated by sodium dodecyl sulfate-polyacrylamide gel electrophoresis (SDS-PAGE) on 10% resolving gels under denaturing and reducing conditions. Separated proteins were electroblotted to polyvinylidene difluorid (PVDF) membranes according to manufacturer’s recommendations (Roche Molecular Biochemicals, Roche Applied Science, Penzberg, Germany). Blots were incubated with antibodies to human CAV-1 (#3238, Cell Signaling Technology, Danvers, MA, USA) and β-actin (monoclonal anti-β-actin, Clone AC-74, Nr. A5316, Sigma-Aldrich Chemie GmbH, Steinheim, Germany) for 16 h at 4°C. After washing in Tris-buffered saline containing 0.1% Tween 20 (TBST, pH 7.4), blots were incubated for 30 min at room temperature with anti-IgG secondary antibody conjugated to horseradish peroxidase. After washing in TBST, bands were visualized by the Amersham ^TM^ ECL^TM^ Prime Western Blotting Detection Reagent (GE Healthcare, Chicago, IL, USA). To confirm the specificity of the antibody, the immunoblotting was repeated independently with a second anti-human caveolin-1 antibody (AF5736 human caveolin-1 antibody AF 5736, R&D Systems, Bio-Techne Corporation, Minneapolis, MN, USA).

### Statistical analysis

As a pilot study, the data was descriptively analyzed and shown as box plots and bar plots, using the GraphPad Prism software 6 for Windows, Version 6.01 (GraphPad Software, La Jolla, CA, USA). Due to the pilot character of the study, *p*-values were regarded as a descriptive analysis. The statistical program IBM SPSS version 23.0 (IBM, Armonk, NY, USA) was used for the statistical evaluation of the data, which was performed in cooperation with the Institute for Medical Biometry Epidemiology and Computer Science (IMBEI) of the University Medical Center Mainz. The Wilcoxon test was used to test for significant differences in Cav-1α and Cav-1β in CAFs and in NFs. The Kruskal-Wallis test and the Mann-Whitney *U* test were used to test for significant differences in Cav-1 expression in CAFs of different tumor stages.

## Results

### Patient collective

For this study, twenty OSCC patients, 13 males and 7 females, ranged from 47 to 92 years old, who underwent tumor resection at the Department of Oral and Maxillofacial Surgery - Plastic Surgery at the Johannes Gutenberg-University of Mainz were enrolled. From these patients, a tumor and a mucosal biopsy were taken. The tumors of the patients were classified according to the TNM classification as follows: 3× the tumor stage T1, 6× the tumor stage T2, 2× the tumor stage T3, 9× the tumor stage T4, 9× the lymph node status N0, 5× the lymph node status N1, 4× the lymph node status N2b, and 2× the lymph node status N2c and 20× assigned to remote metastasis M0 (Table 1). In cell culture, one tumor biopsy and two mucosal biopsies failed to grow. Therefore, CAFs were grown from 19 tumor biopsies and NFs from 18 mucosal biopsies.

**Table 1:** Patient collective

Characteristics	No. of patients (%)
Age, years	
Mean (range)	63.35 (42-87)
Sex	
Male	13 (65)
Female	7 (35)
Primary site	
Tongue	7 (35)
Oral floor	9 (45)
Buccal mucosa	2 (10)
Palate	2 (10)
pT classification	
T1	3 (15)
T2	6 (30)
T3	2 (10)
T4	9 (45)
pN classification	
N0	9 (45)
N1	5 (25)
N2	6 (30)
pM classification	
M0	20 (100)

### Gene expression of the two isoforms in CAFs and NFs

**Fig. 2:**
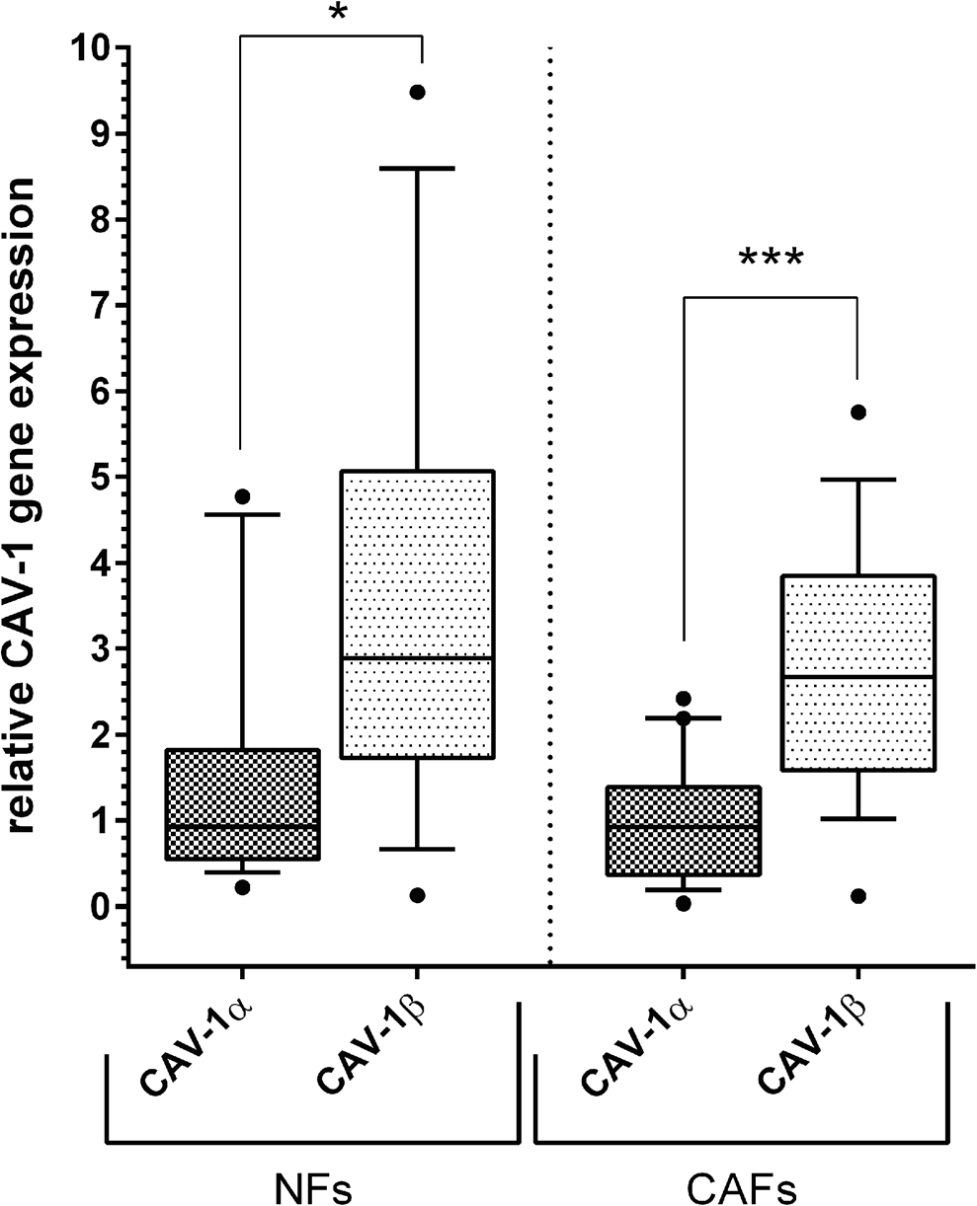
Relative expression of the mRNA of the Cav-1 isoforms Cav-1α and Cav-1β in NFs (left) and in CAFs (right). In normal fibroblasts as well as in cancer-associated fibroblasts of the same patients, CAV-1β expression was more pronounced than CAV-1α expression. Data is shown as box and whisker plots with median and 10 to 90 percentiles, outliers are shown as dots, and Wilcoxon test was used for comparison of isoform expression between NFs (*n* = 18) and CAFs (*n* = 19), * *p* < 0.05, ** *p* < 0.01, *** *p* ≤ 0.001

Primary CAFs (*n* = 19) and NFs (*n* = 18) from patients with OSCC who underwent tumor resection were isolated. We analyzed the expression of Cav-1α and Cav-1β in CAFs and NFs and found that both were positive for the two isoforms of Cav-1. Independent of the type of fibroblasts, the expression of Cav-1β was higher compared to Cav-1α in CAFs (*p* < 0.001) and in NFs (*p* = 0.018) (Table 2, Fig. 2).

**Table 2:** Comparison of the mRNA expression between the Cav-1 isoforms in NFs and CAFs

		Cav-1α	Cav-1β	*p*
CAFs	Median	0.92	2.66	*p* < 0.001
(*n* = 19)	(Q1; Q3)	(0.36; 1.38)	(1.58; 3.84)	
NFs	Median	0.92	2.88	*p* = 0.018
(*n* = 18)	(Q1; Q3)	(0.54; 1.82)	(1.73; 5.06)

### Gene expression of Cav-1α and Cav-1β in relation to the tumor size

**Fig. 3:**
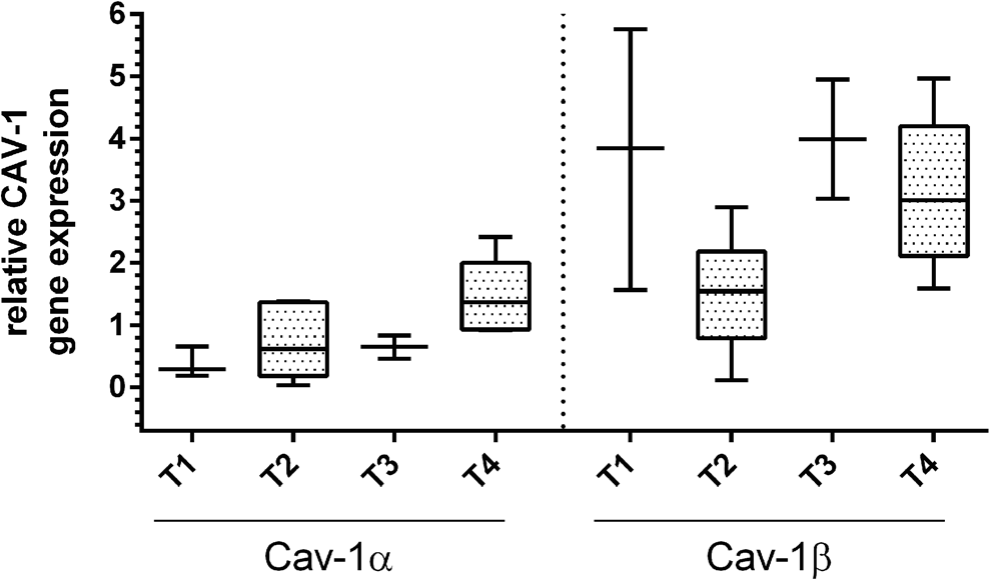
Expression of mRNA of Cav-1α and Cav-1β in the CAFs, divided into the tumor stages T1 to T4. The expression of the Cav-1α isoform was lower than the expression of Cav-1β trough all tumor stages. With higher tumor stages, there was a slight trend for higher Cav-1α expression, while Cav-1β expression was heterogenous throughout the different tumor stages. Data is shown as box and whisker plots with median and 10 to 90 percentiles, and Kruskal-Wallis test was used for comparison between expression in CAFs of different tumor stages, *n* = 19. * *p* < 0.05, ** *p* < 0.01, *** *p* ≤ 0.001

Relating the expression of the two isoforms of Cav-1 in the CAFs to the tumor stages of the patients, they showed an increased Cav-1α expression associated with an advanced tumor stage (*p* = 0.007, Table 3). In comparison to tumor stage T1 (*n* = 3), the highest expression of Cav-1α was found in tumor stage T4 (*n* = 8) (*p* = 0.008, Table 3, Fig. 3). This phenomenon could not be seen comparing Cav-1β expression levels with the tumor stages. In the different tumor stages, heterogeneous expression levels of Cav-1β were shown (*p* = 0.041, Table 3, Fig. 3). The lowest expression was found in stage T2 (Table 3, Fig. 3). Looking at the expression levels in the advanced tumor stages, it is noticeable that the expression of Cav-1β by CAFs (*p* = 0.016) was significantly higher compared to the Cav-1α isoform.

**Table 3:** Expression of the mRNA of the isoforms in CAFs, divided into different tumor stages T1 to T4

Comparison between expression of Cav-1α in CAFs of different tumor stages						
		T1 (*n* = 3)	T2 (*n* = 6)	T3 (*n* = 2)	T4 (*n* = 8)	*p*
Cav-1α	Median (Q1; Q3)	0.29 (0.19; 0.65)	0.62 (0.18; 1.37)	0.65 (0.46; 0.83)	1.37 (0.93; 2.00)	*p* = 0.007
Pairwise comparison of Cav-1α in different tumor stages						
	T1 - T3	T1 - T2	T1 - T4	T3 - T2	T3 - T4	T2 - T4
Cav-1α	*p* = 0.604	*p* = 0.379	*p* = 0.008	*p* = 0.856	*p* = 0.092	*p* = 0.028
Comparison between expression of Cav-1β in CAFs of different tumor stages						
		T1 (*n* = 3)	T2 (*n* = 6)	T3 (*n* = 2)	T4 (*n* = 8)	*p*
Cav-1β	Median (Q1; Q3)	3.84 (1.56; 5.75)	1.54 (0.79; 2.18)	3.99 (3.03; 4.94)	3.01 (2.11; 4.20)	*p* = 0.041
Pairwise comparison Cav-1β in different tumor stages						
	T1 - T3	T1 - T2	T1 - T4	T3 - T2	T3 - T4	T2 - T4
Cav-1β	*p* = 1.00	*p* = 0.167	*p* = 0.776	*p* = 0.071	*p* = 0.533	*p* = 0.020
Comparison between expression of Cav-1α and Cav-1β in CAFs of tumor stages T4 (*n* = 8)						
		Cav-1α	Cav-1β	*p*		
	Median (Q1; Q3)	1.37 (0.93; 2.00)	3.01 (2.11; 4.20)	*p* = 0.016

### Protein expression analysis of Cav-1 in CAFs and NFs

**Fig. 4:**
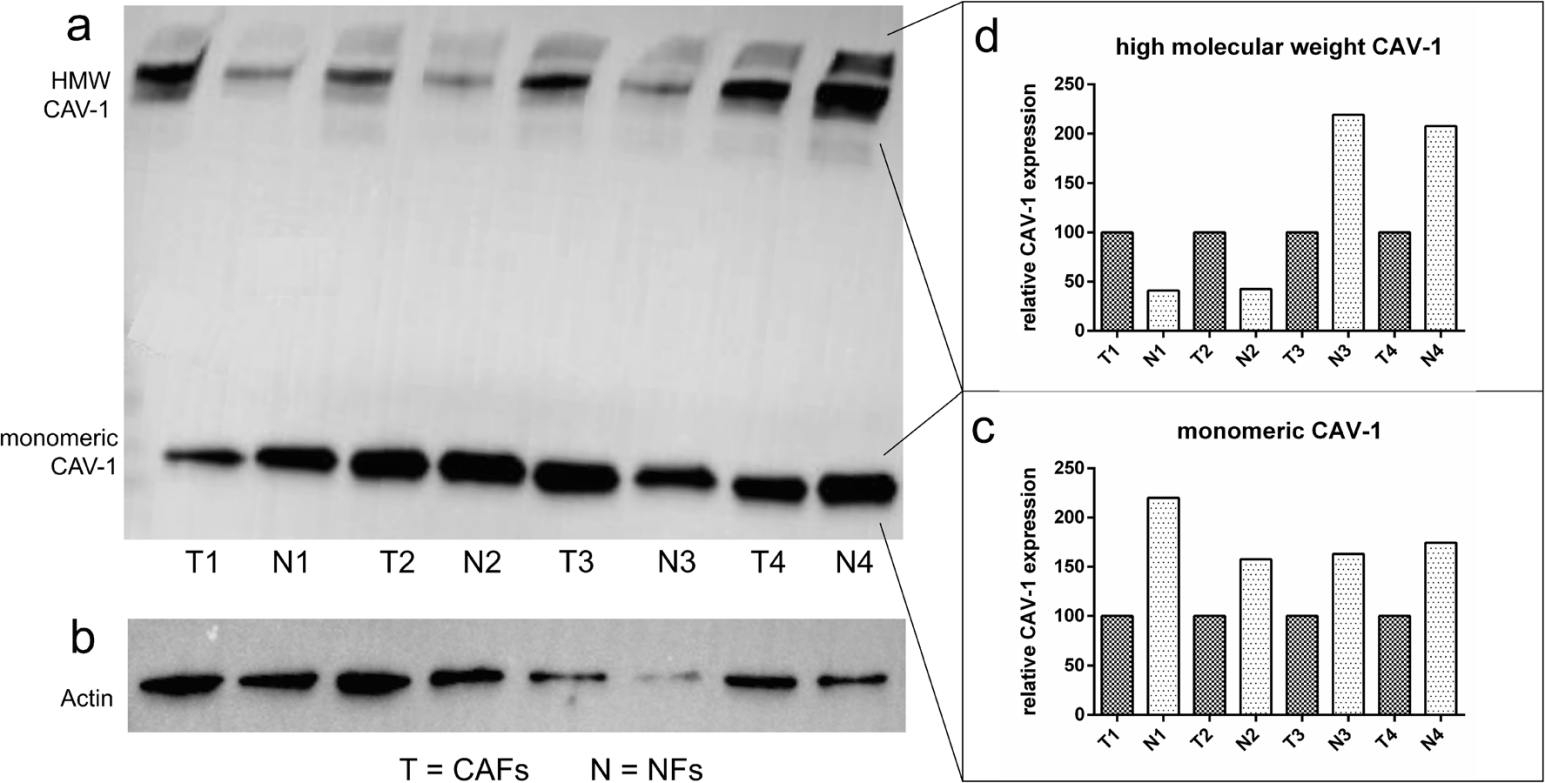
Protein expression analysis of Cav-1 in CAFs and NFs by western blotting. Here an exemplary western blot of lysates from NFs and CAFs is shown. Probes from the same patient were loaded next to each other onto the gel (T = fibroblasts for tumor tissue, N = fibroblasts from normal tissue; T1 and N1 originate from the same patient). Cav-1 could be detected as monomeric protein and as high-molecular weight fraction (**a**). As a reference protein, beta-actin was chosen (**b**). Cav-1 expression is presented in two different values, one for the monomeric form (**c**) and one for the high-molecular weight fraction (**d**). Each Cav-1 expression value was normalized with beta-actin and the paired samples from the same patient were expressed in relation to each other. This means Cav-1 expression in CAFs (= 100%) was related to Cav-1 expression in NFs of the same patient. Cav-1 expression varied between different patients in NFs and in CAFs (**a**). In relation to the tumor sample, the expression of monomeric Cav-1 was higher in NFs (**c**). In contrast, expression level of the high-molecular weight fraction of Cav-1 was heterogenous, sometimes being higher in CAFs and sometimes higher in NFs (**d**)

Protein expression analysis of Cav-1 via western blotting revealed that expression differed between CAFs and NFs of the same patient (Fig. 4). It was striking that apart from monomeric Cav-1 at about 22 kDa, also a high-molecular weight fraction could be detected at >225 kDa despite the usage of denaturing and reducing conditions. The specificity of the antibody was confirmed by crosschecking with a second antibody obtained from a different company. Expression of Cav-1 differed between CAFs and NFs of the same patient in the monomeric form and also the high-molecular weight fraction (Fig. 4).

## Discussion

Cav-1 is an interesting scaffolding protein, which is involved in caveolae formation in cellular membranes. Previous studies demonstrated that the Cav-1 gene can act on the one hand as an oncogene [31, 32] and on the other hand, it can perform an inhibitory role in oral carcinogenesis [33]. Hung et al. associated the increased expression of Cav-1 in patients with OSCC with tumor development [31] and Nohata et al. as well as Auzair et al. with the poor survival of the patients [34, 35]. In contrast to these findings, others associated a low expression of Cav-1 in tumor cells of squamous cell carcinoma of the head and neck with an increase in cell motility and invasion [13]. None of the studies investigated the two isoforms of Cav-1 in OSCC patients.

Analyzing the expression of the two isoforms of Cav-1 in NFs and CAFs of patients with OSCC, we found a different expression at the mRNA level. The expression of Cav-1β was higher in the CAFs and in the NFs of OSCC patients compared to Cav-1α. Until now, all studies only have considered the total Cav-1 expression in tumors and no other studies exist in the literature on the expression profile of the two isoforms of Cav-1 in tumor cells. Recently, Yamao et al. demonstrated the protein expression of the two isoforms of Cav-1α and Cav-1β in the CAFs from pancreatic carcinoma. However, they only showed the expression of both isoforms, but did not quantify their relative strength of expression, nor their function [36]. Williams et al. showed that growth-stimulating mechanisms can be exercised via certain regions of the Cav-1 structure via tyrosine and serine phosphorylation. Cav-1 thereby can exert its role as a tumor oncogene [11]. Tyrosine kinases are known to play an essential role in regulating the control and differentiation of cell growth [37]. However, tyrosine phosphorylation only occurs at residue 14 (Tyr 14) in the Cav-1α isoform [20, 21]. Moreover, it has been reported that phosphorylation of Cav-1 on tyrosine 14 occurs both in vitro and in vivo. In addition, it has been shown that phosphorylation of Cav-1 functionally increases growth and cell migration [38]. Thus, these interrelations might explain why the expression of the Cav-1α isoform might be increased in the tumor microenvironment in comparison to normal mucosa. These aspects show the relevance of further investigations on the two isoforms.

The association between the levels of Cav-1 expression and clinicopathologic factors in this study showed a lower Cav-1α expression compared to the expression of Cav-1β through all tumor stages. With higher tumor stages, there was a slight trend for higher Cav-1α expression, while Cav-1β expression was heterogeneous throughout the different tumor stages. There are no studies showing the expression of isoforms of Cav-1 in relation to tumor stages. However, the total Cav-1 expression was examined in relation to the tumor stages. The results of Xue et al. showed a stepwise increase of Cav-1 expression from normal lining of the tongue to squamous cell carcinoma of the tongue (TSCC). These results underline the possible role of Cav-1 in carcinogenesis and development of TSCC [32].

Comparing the Cav-1 expression in an immunohistochemical examination of biopsies from patients with an oral lichen planus (OLP) with patients with OSCC, an increased Cav-1 expression in OSCC could be detected [39]. Based on this investigation, Cav-1 was assigned a role in the pathogenesis of OLP and OSCC [39]. Huang et al. could not find a correlation between the Cav-1 expression and the tumor stage of OSCC, but they only considered the total Cav-1 expression and did not break it down to the different isoforms [9]. Furthermore, the total expression in the tumor was assessed without analyzing which cells were the origin of the expression.

In the present study, we also evaluated the protein expression of Cav-1 in CAFs and in NFs. Using western blotting, low and high-molecular weight Cav-1 was observed. Interestingly monomeric Cav-1 was decreased in CAFs in relation to NFs of the same patient. This is in line with literature, where expression of Cav-1 is described to be decreased in CAFs in comparison to normal fibroblasts. CAFs have the ability to prevent cancer cell apoptosis, enhance the proliferation of cancer cells, and stimulate tumor angiogenesis [40]. In various tumor entities such as breast cancer [41], gastric cancer [42], and prostate cancer [43], downregulation of Cav-1 protein expression in CAFs could be shown. The downregulated Cav-1 protein expression in these tumors was associated with a hyperproliferative CAFs and Cav-1 is a well-known marker of oncogenic transformation in fibroblasts [40]. The protein expression profile of the high-molecular weight fraction of Cav-1 was inhomogeneous. However, all samples had in common that the amount of Cav-1 in CAFs differed from the one in NFs of the same patient. Most likely, the high-molecular weight fraction of Cav-1 in the western blot stems from glycosphingolipids, which were not resolved during the procedure. The interactions between glycosphingolipids and Cav-1 could be a possible explanation for the fact that the protein could be detected not only at a low-molecular weight but also at a high-molecular weight fraction. Glycosphingolipids are involved in the regulation for Cav-1-mediated biological events, such as the regulation in terms of activity and cell surface concentration of membrane receptors [44]. On the molecular level, the association between glycosphingolipids and Cav-1 mediates the oligomerization of the protein and thus might transduce several membrane-associated functions which might also be relevant for the tumor microenvironment [45]. The observed differences in the high-molecular weight fraction of Cav-1 between CAFs and NFs of the same patient could indicate a possible role of Cav-1 oligomerization for malignant transformation. Thus, this aspect should be further analyzed.

## Conclusion

We were able to show that the two isoforms of Cav-1 were differentially expressed in NFs and in CAFs at the mRNA level. Furthermore, we found that paired samples of NFs and CAFs from the same patient differed in their Cav-1 protein level in the monomeric form and in the high-molecular weight fraction. This indicates the importance of the protein for the development of tumors and the relevance of the ratio between the isoforms and their oligomerization for the tumor microenvironment. One strength of the study is that two samples were taken from the same patient, one from the tumor and one from clinically healthy mucosa. This means that interindividual differences in the basic expression level of Cav-1 could not bias the results. At the same time, however, we had to take the risk that the fibroblasts in the clinically apparently healthy mucosa of tumor patients were already altered, possibly not only in the area next to the tumor but also in the entire oral cavity. This interesting question should be investigated in further studies, as it could also be of therapeutic relevance. Currently, there is no answer as to how far the tumor environment extends in OSCC and what therapeutic consequences need to be drawn subsequentially, also regarding the high recidivation rates. Due to the pilot character of the study, only 20 patients could be included, and for sure a larger sample size would help to draw further conclusions concerning the importance of the two different Cav-1 isoforms in CAFs and NFs.

The differences found in the expression of the two isoforms of Cav-1 might account for the inhomogeneous data concerning the role of caveolin-1 in tumor progression. Further research could include a larger patient cohort to disentangle the function of the two different isoforms of Cav-1 as important structural components of caveolae formation and their effects on tumor progression.
